# Exploring disease interrelationships in patients with lymphatic disorders: A single center retrospective experience

**DOI:** 10.1002/ctm2.760

**Published:** 2022-04-22

**Authors:** Stanley G. Rockson, Xin Zhou, Lan Zhao, Davood K. Hosseini, Xinguo Jiang, Andrew J. Sweatt, Dongeon Kim, Wen Tian, Michael P. Snyder, Mark R. Nicolls

**Affiliations:** ^1^ Stanford Center for Lymphatic and Venous Disorders Division of Cardiovascular Medicine Stanford University School of Medicine Stanford California USA; ^2^ Department of Genetics Stanford University School of Medicine Stanford California USA; ^3^ Veteran Affairs Palo Alto Health Care System Palo Alto California USA; ^4^ Division of Pulmonary, Allergy, and Critical Care Medicine Stanford University School of Medicine Stanford California USA

**Keywords:** co‐morbidity, disease co‐occurrence, disease interrelationship, lipedema, lymphedema, lymphovascular disease

## Abstract

**Background:**

The lymphatic contribution to the circulation is of paramount importance in regulating fluid homeostasis, immune cell trafficking/activation and lipid metabolism. In comparison to the blood vasculature, the impact of the lymphatics has been underappreciated, both in health and disease, likely due to a less well‐delineated anatomy and function. Emerging data suggest that lymphatic dysfunction can be pivotal in the initiation and development of a variety of diseases across broad organ systems. Understanding the clinical associations between lymphatic dysfunction and non‐lymphatic morbidity provides valuable evidence for future investigations and may foster the discovery of novel biomarkers and therapies.

**Methods:**

We retrospectively analysed the electronic medical records of 724 patients referred to the Stanford Center for Lymphatic and Venous Disorders. Patients with an established lymphatic diagnosis were assigned to groups of secondary lymphoedema, lipoedema or primary lymphovascular disease. Individuals found to have no lymphatic disorder were served as the non‐lymphatic controls. The prevalence of comorbid conditions was enumerated. Pairwise co‐occurrence pattern analyses, validated by Jaccard similarity tests, was utilised to investigate disease–disease interrelationships.

**Results:**

Comorbidity analyses underscored the expected relationship between the presence of secondary lymphoedema and those diseases that damage the lymphatics. Cardiovascular conditions were common in all lymphatic subgroups. Additionally, statistically significant alteration of disease–disease interrelationships was noted in all three lymphatic categories when compared to the control population.

**Conclusions:**

The presence or absence of a lymphatic disease significantly influences disease interrelationships in the study cohorts. As a physiologic substrate, the lymphatic circulation may be an underappreciated participant in disease pathogenesis. These relationships warrant further, prospective scrutiny and study.

## Graphical abstract

1. Patients from the Stanford Center for Lymphatic and Venous Disorders were clinically assessed for the presence/absence of lymphatic disorders.
2. All relevant comorbid ICD‐10 diagnoses were tabulated to permit statistical analysis.
3. The study disclosed that the presence of lymphatic dysfunction alters disease interrelationships.
4. Future prospective study may provide novel concepts regarding disease pathogenesis and targeted molecular therapeutics.

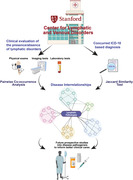

## 1 INTRODUCTION

Disease–disease interrelationships can be considered to reflect the likelihood of co‐occurrence among multiple chronic or acute conditions. These non‐random disease interrelationships theoretically reflect an underlying etiology or pathogenesis shared among the co‐occurring diseases. Disease interrelationships will impact both morbidity and mortality, and comprehension of these interrelationships may substantially influence current and future clinical management. In this context, there is increasing recognition of the lymphatic contribution to the pathogenesis of a wide array of human pathologies, including metabolic, gastrointestinal, neurological, and ocular disorders; neoplasia; and, notably, cardiovascular diseases.^1^, ^2^, ^3^, ^4^, ^5^, ^6^, ^7^ To date, the presence and significance of a lymphatic substrate in given organ‐specific or systemic diseases has been ascertained primarily through pre‐clinical experimental observations. How lymphatic disorders contribute to non‐lymphatic morbidity, and the relevant mechanistic relationships, remain unknown. Enhanced insights into the association of human clinical lymphatic dysfunction with altered disease interrelationships would provide valuable, hypothesis‐generating evidence for future investigation and the potential discovery of novel biomarkers and therapies.

The most common lymphatic circulatory disorder is lymphedema, classified as either primary, due to hereditary or other unidentified in‐born features, or secondary, caused by lymphatic injury originating from infection, cancer, cancer‐related therapies, physical trauma or sustained increases in lymphatic preload.^8^, ^9^ Throughout the world, secondary lymphoedema is the predominant form of the disease. In the US, secondary lymphoedema arises most commonly in cancer patients subjected to lymphadenectomy and/or radiotherapy.^9^ The incidence of secondary lymphedema in cancer patients ranges between 8% and 30%, and the natural history of lymphedema onset within these susceptible subgroups appears to vary significantly.^10^ Risk factors for secondary lymphedema include: obesity at the time of cancer diagnosis, type of surgery, number of lymph nodes removed, radiotherapy protocol and the use of certain chemotherapeutic agents.^10^ Lipedema, another commonly encountered disorder, is a disease characterised by disproportionate, pathological subcutaneous adipocyte hypertrophy that occurs in the context of subclinical compromised lymphatic function.^11^, ^12^, ^13^, ^14^, ^15^, ^16^ Lymphovascular disease, as a subgroup, includes not only primary lymphedema, but also lymphatic malformations, both simple and complex.

We posited that the presence of a lymphatic disease would alter comorbid disease interrelationships. To explore this hypothesis, we performed a retrospective, 3‐year observational study of consecutive, newly evaluated patients in the Stanford Center for Lymphatic and Venous Disorders, a specialized, tertiary care center that is devoted to the evaluation and management of the broad array of lymphatic disease. Our study cohort was comprised of patients with either secondary lymphedema, lipedema or lymphovascular disease. A control comparator group was comprised of those patients consecutively evaluated in the same clinical setting in whom, after thorough evaluation, no lymphatic dysfunction was detected.

To assess comorbidity, we enumerated the prevalence of each of the identified comorbid diagnoses within the studied patient cohorts and performed analyses of disease proportion, risk factors and odds ratios (ORs). To detect the impact of a lymphatic diagnosis upon disease interrelationships, we undertook both disease‐pair co‐occurrence and Jaccard similarity analyses, both of which evaluate pairwise co‐occurrence relationships within binary presence–absence data.^17^ In this single‐center cohort, the presence of a recognized lymphatic disease conferred specific patterns of comorbidity and altered disease interrelationships. The results support the concept that lymphatic function impacts the manner in which distinct conditions arise together; in other words, how these diseases interrelate, presumably on the basis of a heretofore unrecognised, shared dependence upon a lymphatic substrate in pathogenesis. Our findings underscore the potential impact of this lymphatic substrate upon disease expression across a variety of organ systems.

## 2 METHODS

### 2.1 Study design

This retrospective study was conducted at a single academic site (Stanford University), entirely within the Stanford Center for Lymphatic and Venous Disorders, a dedicated lymphatic disease program (https://stanfordhealthcare.org/medical‐clinics/center‐lymphatic‐venous‐disorders.html). The protocol was approved by the Administrative Panels for the Protection of Human Subjects of Stanford University (IRB 64647). Investigations were conducted according to Declaration of Helsinki principles.

Seven hundred and twenty‐four patients who received an initial outpatient clinical evaluation at the center were retrospectively evaluated. Body mass index (BMI) and age were recorded on the date of the initial clinical visit at the center. The presence or absence of a lymphatic disease was ascertained by a lymphatic vascular specialist on clinical grounds, supported by imaging and other clinical analysis, and, in each subject. The presence of any comorbid diagnosis was established through ICD‐10 diagnosis codes assigned within the electronic medical record (Table S1). Each patient in this investigation was assigned either to the non‐lymphatic control group or to the lymphatic cohort. Individuals were assigned to the non‐lymphatic control cohort if, after thorough clinical evaluation, there was no clinical evidence of edema or of any detectable lymphatic substrate to the patient's clinical presentation. A lymphatic diagnosis was sub‐classified into one of three broad categories of lymphatic diseases: secondary lymphedema, lipedema or lymphovascular disease (i.e., primary lymphoedema and/or lymphatic vascular malformation). Note that BMI > 30 is considered as obesity.

### 2.2 Disease incidence

Incidences of a total of 124 conditions were calculated. We followed previously established methodology^18^, ^19^ to use a frequency of 2% as a cut‐off, given median incidences for control and lymphatic cohorts are 0.085 and 0.055.

### 2.3 Comorbidity prevalence

ORs and 95% confidence intervals (CIs) were estimated by logistic regression models using the generalized linear model (glm) function in R. Age, gender and race were considered as confounding variables when calculating the adjusted ORs. Fisher exact tests were performed to calculate the *p*‐values. Diseases with significantly differing proportions between patient cohorts were selected for further investigation. Relative proportions within each cohort were calculated, and the heatmap was created with the ‘ggplot2’ (v3.3.5) package in R.

### 2.4 Disease pairwise co‐occurrence analysis using a probabilistic model

A probabilistic model to study disease co‐occurrence was employed for this investigation.^20^ The model predicts the probability that two diseases would co‐occur at a frequency less than, greater than, or approximately equal to the observed frequency if the two diseases are distributed independently of one another among groups of individuals. Based on the observed and expected frequencies, the model can be used to classify disease associations as negative, positive or random. Positive associations were defined as those in which the observed frequency is significantly greater than expected. Negative associations were defined by an observed frequency less than expected. A random association was iterated for an observed frequency not significantly different from the expected frequency. Disease pairs with less than 1 co‐occurrence were filtered from the ensuing analysis (default value). In this investigation, the presence–absence data were used to test whether pairwise interactions between diseases were random or non‐random using the R library ‘co‐occur’ (v1.3). Significantly non‐random interactions (*p* < .05) were determined via default model specifications. Species pairs that did not occur more than once were excluded (thresh = TRUE). The *p*‐values derived from the probabilistic model are exact probabilities (*p*‐values), which are distribution‐free and non‐parametric (non‐distributional), without reference to a statistic. As this type of probabilistic approach has very low Type I (false positive) and Type II (false negative) error rates due to its lack of reliance on computer algorithm‐based randomization, no *p*‐value corrections were made for multiple comparisons.

### 2.5 Jaccard analysis

For each patient subject, the comorbid ICD‐10 diagnosis codes were tabulated. The data were utilised to calculate the Jaccard index, a statistical parameter that gauges pairwise similarity of disease pairs within a sample set. The Jaccard index is based upon the Jaccard coefficient, which measures the similarity between finite sample sets. The Jaccard coefficient, defined as the size of the intersection divided by the size of the union of the sample sets, can be conceptualized more simply as the number of patients with co‐occurrences of disease A and B divided by the sum of total occurrences of disease A and B in the entire population (Figures S2 and S3). A Jaccard distance <1 permits the identification of a co‐occurring disease pair that does not display co‐occurrence in the alternate cohort, where the Jaccard distance is equal to 1. The Jaccard analysis detects similarity or dissimilarity of two entities in terms of their context within a matrix of subjects, whereases traditional epidemiolocal methods only consider positive/negative correlations between two signals. Using identical Jaccard algorithms, we first calculated Jaccard index for the lymphatic and non‐lymphatic control patient population and built disease dissimilarity networks. The two networks generated by this computational methodology were utilised to create dendrograms of disease interrelatedness. Statistical comparisons between two dendrograms were made by Mantel tests. *p*‐Value less than .05 is considered as significantly different.

### 2.6 Statistical methods

All computational analyses were generated with R version 4.0.0. The Jaccard distance matrices of pairwise disease distances^21^ were generated in the R package ‘vegan’ (v2.5‐7).^22^ To determine if two distance matrices are statistically similar, a two‐sided Mantel test was performed using R package ‘ape’ (v5.5), and this test was permutated 1000 times to improve the precision of test results.^23^, ^24^ The phylogenic dendrograms of disease networks were generated in the R package ‘factoextra’ (v1.0.7).^25^ All other figures were generated with the R package ‘ggplot2’ (v3.3.5).^26^

### 2.7 Data sharing

The data that support the findings of this study are available from the corresponding authors upon reasonable request.

## 3 RESULTS

### 3.1 Cohort characteristics

Patients in this investigation were assigned either to the non‐lymphatic control group (without a lymphatic abnormality) or to the lymphatic cohort (with an assigned lymphatic diagnosis). The lymphatic patients comprise those with either secondary lymphedema, lipedema or lymphovascular disease. The demographic characteristics of the study population are summarized in Table 1. The control group (*N* = 106) does not differ substantially from the three subgroups of lymphatic patients (*N* = 618) by age, sex, race or BMI. As expected, lymphatic patient cohorts are predominated by female subjects; in particular, lipedema is known to be virtually absent in male patients. Within our retrospective study, the control cohort was also predominantly female. Furthermore, among the secondary lymphoedema patients, the percentage of those with cancer was higher than that either in the lipedema or lymphovascular groups (Table 1), consistent with the fact that cancer and cancer‐related therapies are the predominant causes of secondary lymphoedema in the US.^9^

**TABLE 1:** Patient demographics

		Lymphatic patients (*n* = 618)			
	Control patients (*n* = 106)	Secondary lymphoedema (*n* = 407)	Lipoedema (*n* = 93)	Lymphovascular disease (*n* = 118)	*p*‐Value^a^
Age	53 ± 13	62 ± 15	57 ± 13	45 ± 23	1.2e‐08
Sex (%)					2.6e‐11
Male	7	21	0	37	
Female	93	79	100	63	
Race (%)					3.4e‐05
White	52	53	51	52	
Black	2	3	1	3	
Hispanic	9	6	9	5	
Asian	17	9	2	3	
Other^b^	20	29	37	37	
BMI	28 ± 7	31 ± 10	38 ± 9	30 ± 10	.2

Abbreviation: BMI, body mass index.

^a^ *X* ^2^ test.

^b^ Other include Native American, Pacific Islander and unknown.

### 3.2 Disease incidence within each subgroup

Among the 724 patients enrolled in our study, we observed 114 comorbidities, with an acceptably comparable representation of identified comorbidities among control and lymphatic subjects. Appropriate disease categories use the Medical Subject Heading Controlled vocabulary. To assure that our analysis of disease interrelationships is not distorted by any disproportional disease distribution in each subgroup, we first analysed the prevalence of any comorbid disease within each patient cohort (Table S2 and Figure S1). In the 106 non‐lymphatic control patients, we selected 51 diseases with an incidence greater than 0.02 for analysis. Breast cancer, general anxiety disorder (GAD), chronic back pain, obesity, and hypertension (HTN) are the top five most prevalent conditions (Table S2 and Figure S1). Among 618 lymphatic patients, 66 comorbid conditions surpassed the same incident threshold, with 66 conditions selected in the secondary lymphedema group, 46 selected in the lipedema group and 38 in the lymphovascular disease group (Table S2 and Figure S1). Within the lymphatic cohort, secondary lymphoedema, lipoedema and lymphovascular disease reflect an incidence of 0.66, 0.15 and 0.19, respectively. Other common diagnoses among the lymphatic patients are obesity, HTN, hypercholesterolemia, chronic venous insufficiency (CVI), gastroesophageal reflux disease (GERD), chronic back pain, cellulitis, osteoarthritis, GAD and breast cancer.

### 3.3 Comorbid prevalence within the study cohorts

**FIGURE 1:**
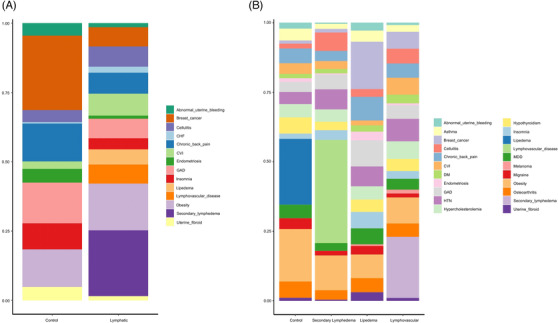
Prevalence distribution of comorbidities by patient cohort. (A) Patients were assigned to either the control group (individuals without a lymphatic diagnosis) or the lymphatic group (subjects with clinically ascertained lymphatic abnormalities). (B) The lymphatic subjects were represented by those with secondary lymphedema, lipedema or lymphovascular disease. Within each study cohort, the prevalence of a given comorbid diagnosis was calculated as the ratio of disease positive subjects to the total number of subjects within the cohort. The composition of these diseases was plotted as their relative ratio to the total disease occurrence (this ratio is normalized to 1 for the purpose of cross‐group comparison)

In order to facilitate the analysis of the most frequently represented comorbid diagnoses within this study population, we calculated and compared disease proportions, as well as relative risks and ORs (Figure 1 and Tables 2, 3 and S3). These analyses highlight the significant association of congestive heart failure (CHF, risk ratio 6.00, 0.83–43.35, *p* = .05) and CVI (risk ratio 3.36, 1.62–6.97, *p* = .0001) (Table 2). The observation of a robust, significant comorbidity among cardiovascular abnormalities and the presence of a lymphatic diagnosis resonates with multiple preclinical studies that underscore the role of the lymphatic vasculature in cardiovascular function and pathology.^5^, ^27^ Obesity is strongly associated with lipedema in comparison with lymphedema and lymphovascular diseases, consistent with the abnormal adipose/lymphatic interactions that are hypothesised for lipedema pathogenesis (Tables 3 and S3). We also documented that venous insufficiency and breast cancer are significant comorbid conditions of secondary lymphedema (Tables 3 and S3). This observation is congruent with the reported high prevalence of breast cancer and venous insufficiency in a recent large clinical study of secondary lymphedema.^28^ Cellulitis, a common source of morbidity in lymphoedema patients,^28^ is demonstrably linked with lymphedema in the current analysis (Tables 3 and S3). GAD is uniquely associated with all lymphatic disorders suggesting a potential role for lymphatic function in the central nervous system (Tables 3 and S3).

**TABLE 2:** Fourteen diseases with significant different proportions between lymphatic and control groups

	Relative proportions			Crude ORs (95% CI)			Adjusted ORs (95% CI)		
	Control	Lymphatic	*p*‐Value	OR	Lower CI	Upper CI	OR	Lower CI	Upper CI
Congestive heart failure	0.40	2.05	.05	6.30	1.34	112.62	4.29	0.87	77.64
Chronic venous insufficiency	2.81	8.01	.00	4.03	1.96	9.74	3.31	1.58	8.09
Cellulitis	4.42	7.43	.02	2.23	1.21	4.53	2.43	1.25	5.23
Obesity	13.65	16.90	.01	1.86	1.21	2.91	1.58	1.00	2.54
Chronic back pain	13.65	7.60	.02	0.56	0.36	0.89	0.53	0.32	0.87
Generalised anxiety disorder	14.86	7.08	.00	0.45	0.29	0.71	0.58	0.36	0.94
Insomnia	9.24	3.98	.00	0.45	0.27	0.77	0.49	0.28	0.86
Abnormal uterine bleeding	4.42	1.35	.01	0.33	0.16	0.73	0.47	0.21	1.07
Uterine fibroid	4.82	1.46	.01	0.33	0.16	0.70	0.36	0.17	0.80
Endometriosis	4.82	1.05	.00	0.24	0.11	0.52	0.25	0.11	0.56
Breast cancer	26.91	6.96	.00	0.14	0.09	0.21	0.15	0.09	0.25

Abbreviations: CI, confidence interval; OR, odds ratio.

**TABLE 3:** Nineteen diseases with significantly different proportions between any of the two groups

	Control	Secondary lymphoedema	Lipoedema	Lymphovascular disease
Obesity	8.61	9.42*	18.93**	12.58
Hypertension	7.09	8.13^*^	4.35	7.23
Hypercholesterolemia	4.81	6.30^*^	4.86	4.40
Chronic venous insufficiency	1.77	6.08**	3.84^*^	2.83
Breast cancer	16.96	5.97**	1.02**	1.26**
Cellulitis	2.78	5.33**	1.79	6.60
Chronic back pain	8.61	5.22	5.37	3.77**
General anxiety disorder	9.37	4.84*	3.58**	5.35**
Osteoarthritis	5.06	4.79	5.88	3.46
Hypothyroidism	4.56	4.47	5.88	3.14
Major depressive disorders	5.82	3.98	4.86	2.83**
Diabetic mellitus	2.28	3.12	1.53	1.57
Insomnia	5.82	2.69^*^	1.79^*^	3.46^*^
Asthma	4.05	2.53	4.35	1.89^*^
Migraine	3.04	1.45	3.84	1.57
Melanoma	0.51	1.29	0.00	0.00
Uterine fibroid	3.04	1.08^*^	1.02	0.31**
Abnormal uterine bleeding	2.78	0.75^*^	2.05	0.31**
Endometriosis	3.04	0.65**	1.28	0.31**

*Note*: Asterisks indicate statistical significance in comparisons between each lymphatic abnormalities with the control cohorts.

* *p* < .05.

** *p* < .01.

### 3.4 Disease pairwise co‐occurrence patterns within patient cohorts

**FIGURE 2:**
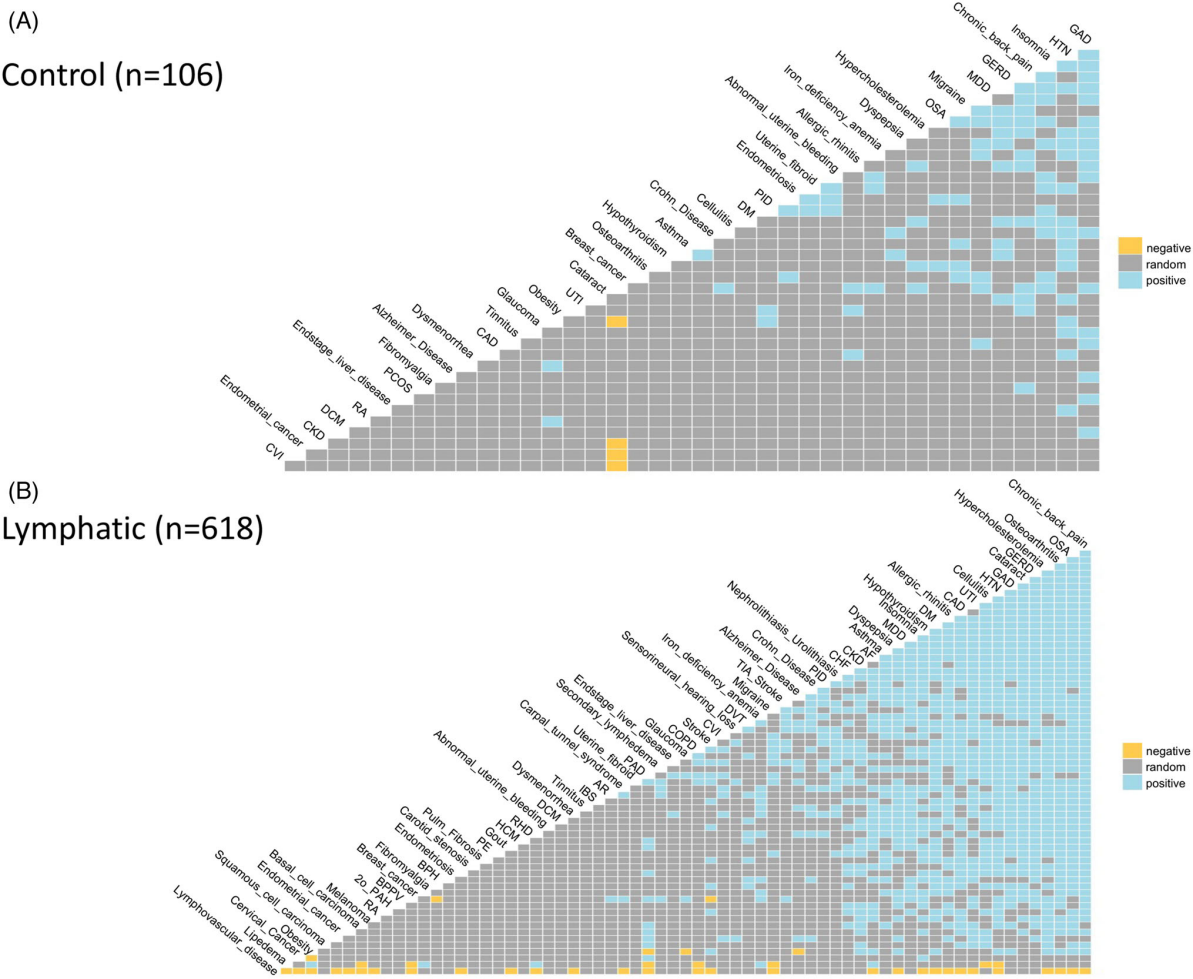
Pairwise co‐occurrence analysis indicates prominent disease interrelationships among lymphatic patients. Disease interrelationships in association with or without ascertained lymphatic diagnosis was calculated by a probabilistic model. Positive associations are defined as those in which the observed frequency is significantly greater than expected one. Negative associations are defined by an observed frequency less than expected. A random association is iterated for an observed frequency not significantly different from the expected frequency. Disease pairs with less than 1 co‐occurrence were filtered from the ensuing analysis

To further understand the potential phenomenon of disease interrelationships in association with a lymphatic diagnosis (as distinct from comorbidity with a lymphatic diagnosis), we further applied a probabilistic model to analyse disease co‐occurrence.^20^ Through this analysis, we observed prominent disease interrelationships among lymphatic patients that were not observed in the control cohort (Figures 2 and S1 and Table 4). Among the identified 1591 disease pairs within the lymphatic patients, we found 943 positive, 42 negative and 606 random associations; correspondingly, there were 106 positive, four negative and 357 random associations within the control cohort.

**TABLE 4:** Numbers of positive, negative and random pairwise disease associations

	Positive	Negative	Random
Lymphatic (*n* = 618)	943	42	606
Secondary lymphedema (*n* = 407)	742	5	711
Lipedema (*n* = 93)	99	0	213
Lymphovascular (*n* = 118)	57	0	94
Non‐lymphatic control (*n* = 106)	106	4	357

**FIGURE 3:**
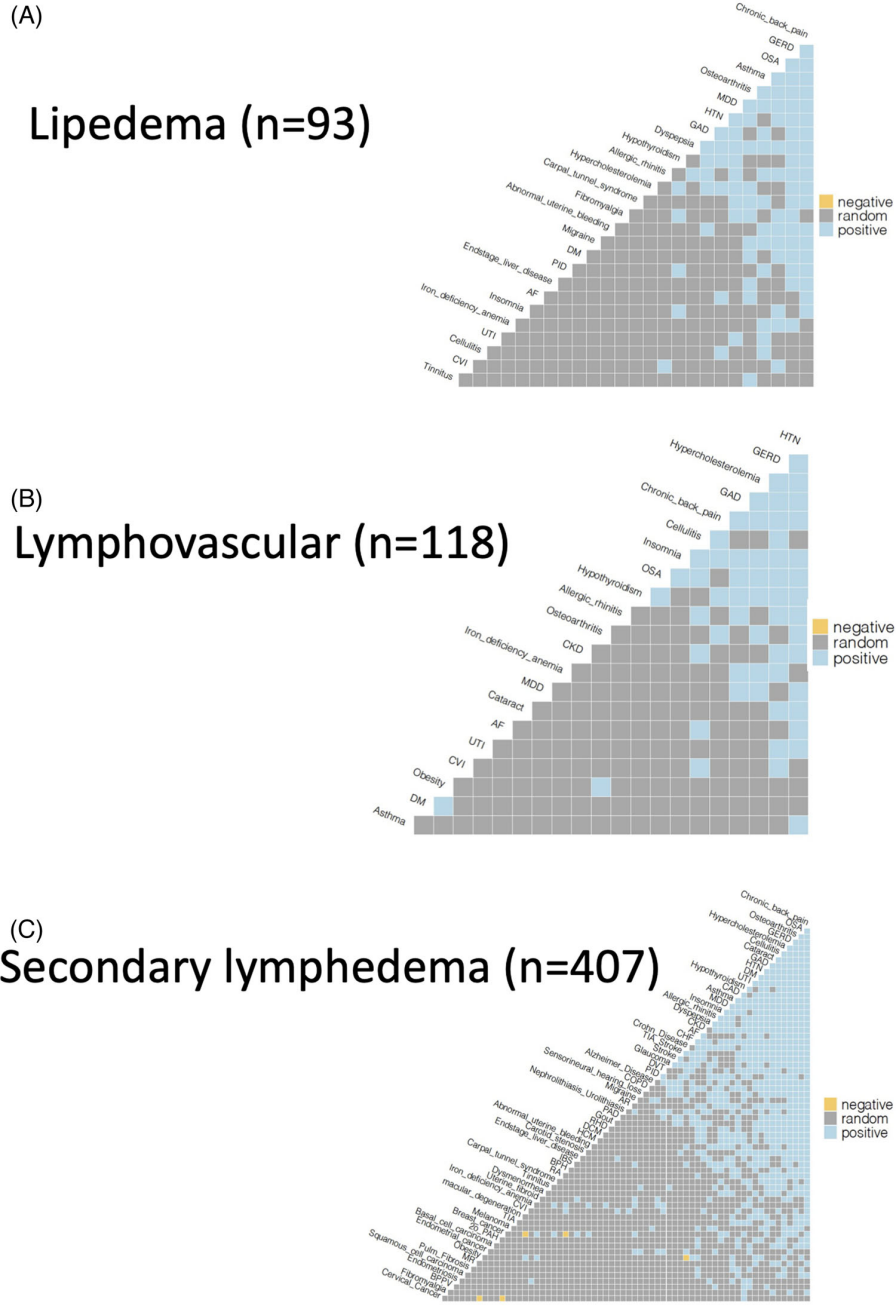
Disease co‐occurrences in three lymphatic subgroups. Detailed analyses of disease co‐occurrences in secondary lymphedema, lipedema and lymphovascular disease groups

Detailed analysis of the three lymphatic subgroups subsequently identified 1458, 312 and 151 disease co‐occurrences in secondary lymphedema, lipedema and lymphovascular disease patients, respectively (Figures 3 and S1 and Table 4). Of note, 742 positive, five negative and 711 random associations were found in the secondary lymphoedema patients. Significant disease interrelationships were more frequently encountered in lymphovascular patients than in lipedema patients (312 vs. 151 disease pairs), despite the comparable sizes of the two patient cohorts (93 and 118, respectively).

### 3.5 Jaccard similarity analysis identifies unsuspected disease relationships

Having identified the relative prevalence of disease pairwise occurrence, we next sought to detect and confirm unsuspected clustering among identified comorbidities. Jaccard testing is a powerful and common tool to illustrate human disease network and comorbid profiles.^29^, ^30^, ^31^, ^32^, ^33^ Given the large number of observed comorbidities within the lymphatic patients, we hypothesised that a Jaccard analysis of the aggregated group of comorbidities would offer novel insights into the impact of a lymphatic diagnosis upon any associated comorbid disease interrelationships (specifically, the relative likelihood of any other pairs of diagnoses to be identified within the same patient).

**FIGURE 4:**
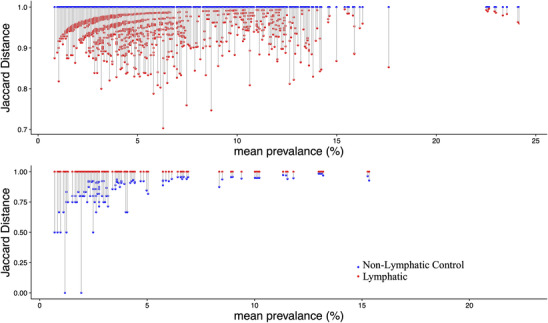
Jaccard similarity analysis of comorbidity: the impact of concomitant lymphatic disease diagnosis. The Jaccard distance is calculated from the Jaccard index (defined in the text). A Jaccard distance of 1 defines a disease pair that does not occur together within a cohort, while a Jaccard distance <1 defines a disease pair that does simultaneously occur. The smaller the magnitude of the Jaccard distance (the longer the line graphically depicted), the more likely it is that the specific disease pair will be detected within a given data set. (Upper panel) Graphical representation of the Jaccard distances for disease pairs that coexist in the lymphatic disease cohort (red) but do not have co‐occurrence in the non‐lymphatic cohort (blue); in the lymphatic disease cohort the prevalence of these disease pairs increases. (Lower panel) A comparable analysis for disease pairs that are represented in the non‐lymphatic cohort (blue) that do not co‐occur in the lymphatic disease cohort (red). For both graphs, the mean disease prevalence is depicted on the *X* axis and the Jaccard distance on the *Y* axis

We calculated 3081 pairs of comorbidities within the control group, and 6670 pairs within the lymphatic cohort. Here, 59.23% of comorbidity pairs in the control cohort and 40.82% pairs in lymphatic cohort have an index of 1 (i.e., no observed co‐occurrence in our data set). We subsequently investigated those comorbidity pairs with a Jaccard index equal to or <1, respectively. Through this analysis, we observed that a large number of the identified disease pairs with an index of 1 (absence of co‐occurrence) in the control group gained co‐occurrence, to varying degrees, in the presence of a lymphatic diagnosis (Figure 4, upper panel and Tables S4 and S5). This gain of co‐occurrence was observed across the spectrum of disease prevalence, from low to high. In contrast, the disease pairs that lose co‐occurrence in the presence of a lymphatic diagnosis (Figure 4, lower panel and Tables S4 and S5) are numerically fewer; these changes are observed predominantly within low prevalence pairs and with small changes in the calculated Jaccard distance. This analysis further suggests that lymphatic disorders promote disease co‐occurrence.

### 3.6 Lymphatic diagnoses influence the relationships among disease conditions

**FIGURE 5:**
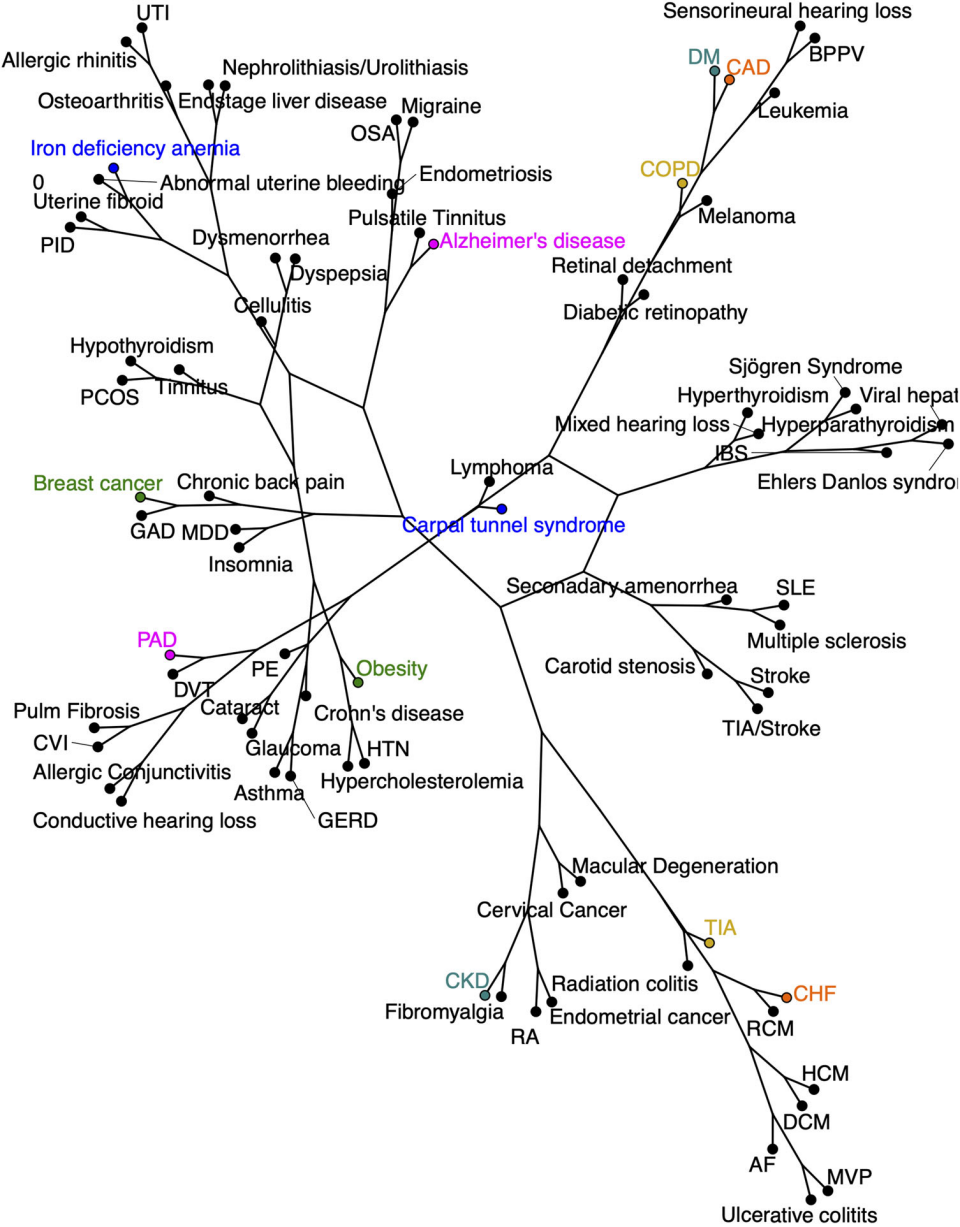
Disease network dendrograms generated by Jaccard similarity analysis: control group. Data from Jaccard similarity analysis were utilized to generate disease network dendrograms for control patients without an ascertained lymphatic diagnosis. Representative disease pairs highlighted here display significant alteration in comparison to the network for lymphatic cohorts in Figure 6. AF, atrial fibrillation; AoAneur, aortic aneurysm; AR, aortic regurgitation; AS, aortic stenosis; BPPV, benign paroxysmal positional vertigo; CAD, coronary artery disease; CF, cystic fibrosis; CHF, congestive heart failure; CKD, chronic kidney disease; COPD, chronic obstructive pulmonary disease; CRVO, central retinal vein occlusion; CVI, chronic venous insufficiency; DCM, dilated cardiomyopathy; DM, diabetes mellitus; DVT, deep vein thrombosis; GAD, generalised anxiety disorder; GERD, gastroesophageal reflux disease; HCM, hypertrophic cardiomyopathy; HTN, hypertension; IBS, inflammatory bowel syndrome; MDD, major depressive disorder; MR, mitral regurgitation; MS, multiple sclerosis; MVP, mitral valve prolapse; OSA, obstructive sleep apnea; PAD, peripheral arterial disease; PAH, pulmonary arterial hypertension; PCOS, polycystic ovary syndrome; PE, pulmonary embolism; PID, pelvic inflammatory disease; PR, pulmonic regurgitation; PS, pulmonic stenosis; RA, rheumatoid arthritis; RHD, rheumatic heart disease; SLE, systemic lupus erythematosus; TIA, transient ischaemic attack; TR, tricuspid regurgitation; UTI, urinary tract infection

**FIGURE 6:**
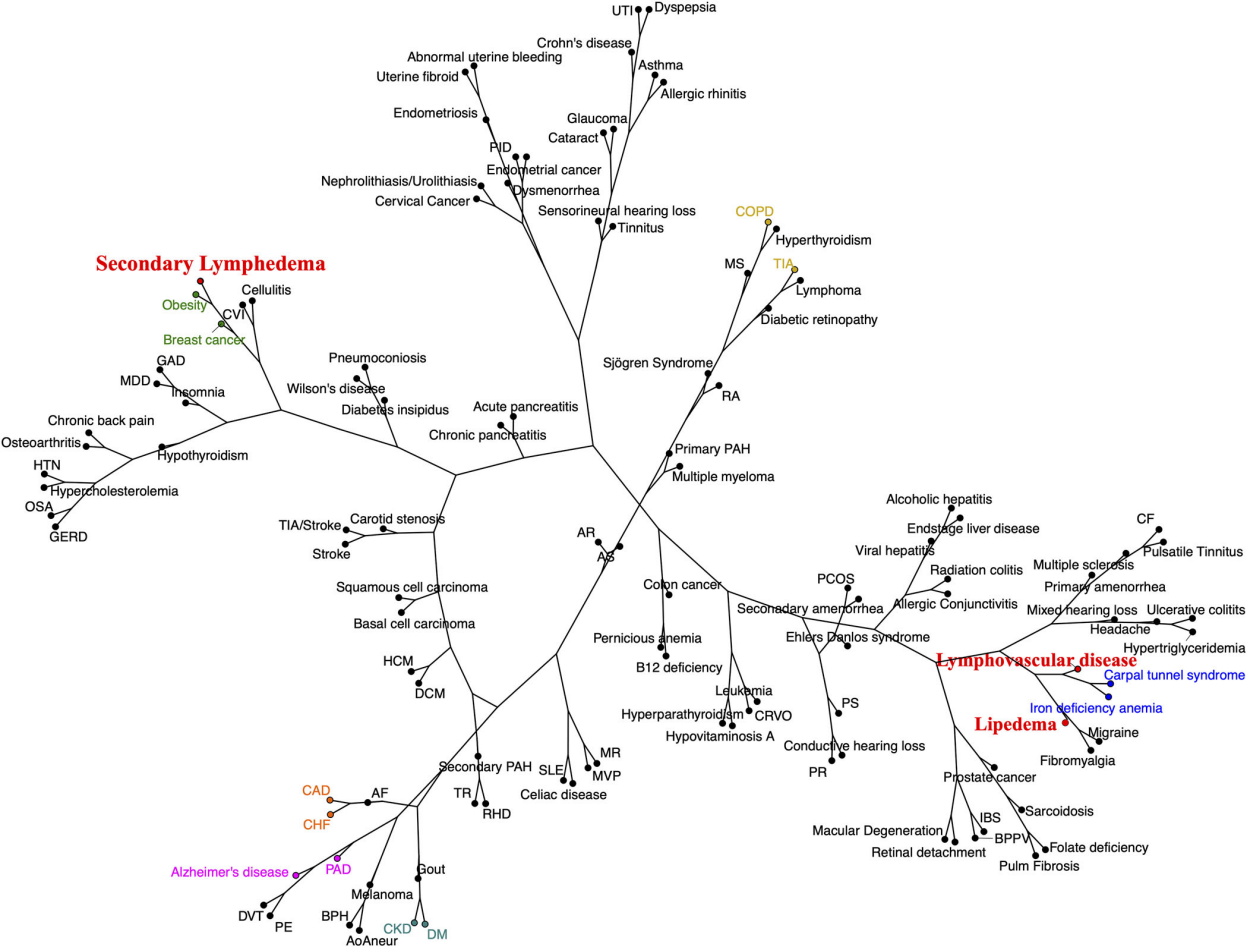
Disease network dendrograms: lymphatic groups. The disease network dendrogram for those with a lymphatic disorder is significantly different thanthe control network in Figure 5 (*p* < .001). The lymphatic network is more expansive, with distinct disease re‐ordering. The three lymphatic disease categories are designated in red. Representative disease pairs are highlighted in identical color schemes as in Figure 5 network, for visual comparison

To facilitate the interpretation of the large number of Jaccard indices generated through this analysis, we decided to graphically depict the indices as distances linking each of the disease pairs within the analysis. Accordingly, we generated dendrograms of disease networks based on the Jaccard distances derived from the calculations. We compared the comorbidity disease network of the lymphatic cohort with that of the control cohort. As expected from the data depicted in Figure 4, the lymphatic dendrogram demonstrates a distinct reordering of disease–disease relationships when compared to the dendrogram of the non‐lymphatic control cohort (Figures 5 and 6). The Mantel statistic indicates that these two pairwise disease distance matrices are highly significantly different (*p* < .001).

Closer examination of the content of these dendrograms discloses the fact that, in comparison with the normal disease cohort, the relationships among specific disease entities are substantially re‐aligned in the presence of a lymphatic diagnosis (Figures 5 and 6). This is true, for example, for the likelihood of concomitant obesity and breast cancer: while these two entities are well recognized to be mechanistically linked,^34^ the linkage between obesity and breast cancer is remarkably closer in the presence of a lymphatic disease than in the non‐lymphatic control patient cohort. These results support the concept that lymphatic function impacts the way distinct diseases arise together and the manner in which these conditions interrelate. Additional examples of disease pair likelihood that is strengthened by the presence of a lymphatic diagnosis include: chronic obstructive pulmonary disease (COPD)/transient ischaemic attack (TIA); CHF/coronary artery disease; chronic kidney disease (CKD)/diabetes mellitus (DM); and peripheral arterial disease (PAD)/Alzheimer's disease (Figure 6 with highlights), among others. In many of these disease pairs, there is a historically recognized mechanistic relationship that is apparently intensified in the presence of a concomitant lymphatic diagnosis; in others, the mechanistic link has not been identified or, in some cases, suspected. By inference, these observations, supported by quantitative analysis, suggest that the presence or absence of lymphatic integrity has the potential to influence the pathogenesis or natural history of pairs of diseases that may not be canonically linked to one another through identified, shared disease mechanisms.

## 4 DISCUSSION

In this clinical investigation, we have retrospectively scrutinized the medical records of a cohort of 724 patients that were clinically evaluated for the presence or absence of a lymphatic diagnosis. This cohort of patients, drawn from the general medical population, was referred to the tertiary care clinic for the specific ascertainment of the presence or the absence of lymphatic pathologies, giventhat this expertise is often underrepresented at the community level. In these patients, consecutively evaluated in our center devoted to the evaluation and management of lymphatic disease, the comorbid disease relationships of the 618 patients with a lymphatic diagnosis were compared with those of the 106 patients in whom, after thorough evaluation, no underlying lymphatic diagnosis could be established. Despite a relatively small sample size, our lymphatic cohort appears to display comorbid characteristics comparable to larger data sets previously collected and analysed in the US.^11^, ^28^ Thus, while the pairwise co‐occurrence and Jaccard analyses performed here are specifically relevant to the patient population of our facility's lymphatic disease center, the apparent congruity of this population with other, more widely distributed patient groups suggests that this analysis might be extrapolated to a larger disease population. Results suggest that the presence of lymphatic dysfunction significantly affects the way that co‐existent conditions arise in individuals.

In our initial analysis of comorbidity through a prevalence description, we determined that breast cancer and venous insufficiency were among the most frequently encountered concomitant diseases in secondary lymphedema (Figure 1 and Table 2), as previously reported.^28^ However, despite the prevalence of identical, compelling risk factors for lymphedema, at best, only 30%–40% of at‐risk individuals develop lymphedema. In this context, one might hypothesize that overt lymphedema after lymphatic vascular injury requires additional ‘hits’ such as genetic mutations that not only limit lymphatic repair and regeneration^35^ but also promote disease in other organ systems. Two conditions characterized by pathological lymphatic remodelling,^4^, ^36^, ^37^ glaucoma and macular degeneration, were represented in our secondary lymphedema cohort. CKD, another co‐existent condition in this study, can also present with pathological lymphatic vasculature.^38^ The observed prevalence of venous insufficiency in this secondary lymphedema cohort is consistent with the fact that chronic venous HTN increases the lymphatic preload and leads to secondary lymphatic failure.^39^ The prevalence of an array of cardiovascular diseases in the lymphatic cohort may be explained through similar hypothesized mechanisms.^5^, ^6^, ^27^ Thus, this study did replicate prior reports of specific pathologies that are identified to be comorbid with lymphatic dysfunction.

To build on this initial assessment, we next evaluated the impact of lymphatic dysfunction on the clustering of concomitant diseases using two bioinformatic tools: a disease pairwise co‐occurrence analysis and a Jaccard analysis. These methodologies are perhaps of greater theoretical interest, insofar as such observations may help to uncover unsuspected, unifying mechanisms that relate to the role of the lymphatic circulation in the concomitant maintenance of health in a variety of organ systems. Both analyses have been successfully used to gauge interrelationships between individuals for biological presence–absence data.^17^, ^32^ Utilizing this approach, we evaluated the interrelationships among 114 comorbid diseases in the lymphatic and control cohorts derived from the patient population of the Stanford Center for Lymphatic and Venous Disorders. Our analysis confirmed the clustering between obesity and breast cancer in the lymphatic cohort, thus correctly underscoring the co‐occurrence of these two conditions in acquired lymphedema, as prior studies have demonstrated.^40^, ^41^, ^42^ Obesity is often considered a risk factor for breast cancer, possibly through the promotion of breast tissue tumorigenesis through obesity‐induced local inflammation.^9^, ^43^ Because inflammation is heavily fostered through mechanisms associated with lymphatic immune traffic, any form of undetected, underlying lymphatic dysfunction or structural abnormality could serve as a potential mechanistic link for the clustering of breast cancer and obesity. Of note, in the control cohort, obesity and breast cancer were less tightly clustered than within the lymphatic cohort.

Additional exploration of the disease network dendrograms generated through this Jaccard analysis revealed additional notable pairs of diseases that display shortened Jaccard distances (i.e., a higher likelihood of concomitant presence) in the lymphatic cohort than those observed in the control cohort for the same pairs of diseases. These findings are particularly notable because many of these identified disease pairs represent conditions with relatively and comparably high prevalence in both cohorts examined. These disease pairs include COPD and TIA, PAD and Alzheimer's disease, and CKD and DM. It is known that COPD involves a dropout of alveolar microvasculature,^44^, ^45^ and COPD does increase the risk of stroke.^46^ These results suggest that lymphatic derangements accompany microvascular disease in these conditions. In the presence of a lymphatic diagnosis, COPD and TIA are much more likely to coexist, as reflected by their proximity in the disease network dendrogram. These observations help to generate the hypothesis that an underlying derangement in lymphatic function may, in parallel, promote disease expression of two diseases that do not share an obvious pathogenesis. A similar hypothetical framework may influence the pairing of PAD and Alzheimer's disease,^47^, ^48^ and of CKD and DM.^1^, ^38^, ^49^ Such notions are not definitive, but the hypothesis‐generating nature of these observations might certainly provide a platform for future mechanistic investigations, both in the prospective identification of relevant biomarkers to aid diagnostic identification, and in the evolution of novel, appropriately targeted therapies.

The interpretation of the current analysis must consider our limited ability to accurately detect a relatively comprehensive array of disease prevalence in this finite dataset. Because both disease‐pair co‐occurrence and Jaccard similarity consider global similarity in presence/absence data, and Jaccard analysis transforms presence/absence into graphical distance, the accurate detection the presence of any disease within the studied population will affect relationships among this disease and its associated comorbidities. Here, we observe that the prevalence of common disorders, such as obesity, is comparable to what is observed in the general population. Nevertheless, with a relatively small sample size, our limited dataset may reflect disease prevalence that differs from that observed in larger, general populations. However, in the absence of large public data bases for lymphatic diseases, this highly enriched study cohort does provide a unique ability to examine disease interrelationships to the extent that they differ from a closely matched control cohort that lacks any detectable lymphatic pathology.

Another theoretical limitation of the approach is that we have limited our analysis to a binary consideration of simple presence/absence of the comorbidities analysed in this patient cohort. However, disease can be characterised not only by its presence/absence, but also, and meaningfully, by its severity. As an example, the diagnosis of obesity for this study relied upon the presence of a BMI >30, but our Jaccard analysis does not discriminate mild from super obesity. It can be anticipated that severity of disease has biological implications that would be relevant in an investigation of the lymphatic contribution to disease interrelationships and should also be the subject of future prospective investigation.

In summary, the current retrospective analysis of a cohort of subjects with and without identified lymphatic pathology relies upon a novel concept. In the past, studies of comorbidity have chiefly relied upon the impact of one variable or disease on the incidence or prevalence of comorbidities. Here, we intend to advance the hypothesis, supported by our data, that the presence or absence of identified lymphatic dysfunction has a potential, defining influence on patterns of disease expression and disease interrelationship. Our investigation has inherent limitations that include the single‐center nature of the study, the relatively small cohort of studies subjects, and the reliance upon historical identification of comorbidities. The current study is not intended to identify specific pathologies that may harbor a lymphatic mechanism, but our observations suggest that future, prospective evaluation of these phenomena may provide new insights into disease pathogenesis and, potentially, create a path to innovative, targeted therapeutics.

## Supporting information

Supplementary Figure 1. Disease interrelationships analysed by pairwise co‐occurrences.

Supplementary Figure 2. Schematic representation of the Jaccard distance calculation as a Venn diagram. The distance between subjects A and B is depicted as one minus the total number of diseases found in both subjects (segment 2 of the Venn Diagram) divided by the total number of diseases found in the two subjects (segments 1 + 2 + 3 in the Venn Diagram).

Supplementary Figure 3. Schematic representation of Jaccard distance analysis. As seen in Figure 2 in the text, Jaccard distances of control group are depicted in blue and Jaccard distances in lymphatic groups are shown in red. A value of 1 (blue) indicates that there is no co‐occurrence of the depicted disease pair in the non‐L cohort. The length of the line that connects the blue circle to the red (value < 1) is representative of the likelihood of co‐occurrence of the disease pair within the L cohort; an increasing length of the line indicates an *increased* likelihood of co‐occurrence. A similar display can be created for the disease pairs in which the Jaccard distance = 1 in the lymphatic cohorts (red), but co‐occur in the control cohorts (blue circle, value < 1), thereby permitting analysis of those disease pairs that have a *decreased* likelihood of co‐occurrence in the setting of a lymphatic diagnosis.

Figures

Supplementary Table 1. ICD‐10 codes used in current study.

Supplementary Table 2. Prevalence of diseases in non‐lymphatic control and lymphatic (lymphoedema, lipoedema and lymphovascular disease) groups.

Supplementary Table 3. *p*‐Values for co‐occurrence analysis for each disease among four study groups.

Supplementary Table 4. Jaccard distances calculated for each disease pairs.

Supplementary Table 5. Prevalence of disease pairs in Jaccard analysis.

## References

[1] Jiang X , Tian W , Nicolls MR , Rockson SG . The lymphatic system in obesity, insulin resistance, and cardiovascular diseases. Front Physiol. 2019;10:1402. 31798464 10.3389/fphys.2019.01402 PMC6868002

[2] Oliver G , Kipnis J , Randolph GJ , Harvey NL . The lymphatic vasculature in the 21(st) century: novel functional roles in homeostasis and disease. Cell. 2020;182:270‐296. 32707093 10.1016/j.cell.2020.06.039 PMC7392116

[3] Vieira JM , Norman S , Villa Del Campo C , et al. The cardiac lymphatic system stimulates resolution of inflammation following myocardial infarction. J Clin Invest. 2018;128:3402‐3412. 29985167 10.1172/JCI97192 PMC6063482

[4] Kim J , Park DY , Bae H , et al. Impaired angiopoietin/Tie2 signaling compromises Schlemm's canal integrity and induces glaucoma. J Clin Invest. 2017;127:3877‐3896. 28920924 10.1172/JCI94668 PMC5617682

[5] Rossitto G , Mary S , McAllister C , et al. Reduced lymphatic reserve in heart failure with preserved ejection fraction. J Am Coll Cardiol. 2020;76:2817‐2829. 33303070 10.1016/j.jacc.2020.10.022 PMC7724570

[6] Itkin M , Rockson SG , Burkhoff D . Pathophysiology of the lymphatic system in patients with heart failure: JACC state‐of‐the‐art review. J Am Coll Cardiol. 2021;78:278‐290. 34266581 10.1016/j.jacc.2021.05.021

[7] Rockson SG . Advances in lymphedema. Circ Res. 2021;128:2003‐2016 . 10.1161/CIRCRESAHA.121.318307 34110905

[8] Brouillard P , Boon L , Vikkula M . Genetics of lymphatic anomalies. J Clin Invest. 2014;124:898‐904. 24590274 10.1172/JCI71614 PMC3938256

[9] Jiang X , Nicolls MR , Tian W , Rockson SG . Lymphatic dysfunction, leukotrienes, and lymphedema. Annu Rev Physiol. 2018;80:49‐70. 29029593 10.1146/annurev-physiol-022516-034008 PMC6434710

[10] Rockson SG , Keeley V , Kilbreath S , Szuba A , Towers A . Cancer‐associated secondary lymphoedema. Nat Rev Dis Primers. 2019;5:22. 30923312 10.1038/s41572-019-0072-5

[11] Buck DW 2nd , Herbst KL . Lipedema: a relatively common disease with extremely common misconceptions. Plast Reconstr Surg Glob Open. 2016;4:e1043. 27757353 10.1097/GOX.0000000000001043 PMC5055019

[12] Lohrmann C , Foeldi E , Langer M . MR imaging of the lymphatic system in patients with lipedema and lipo‐lymphedema. Microvasc Res. 2009;77:335‐339. 19323976 10.1016/j.mvr.2009.01.005

[13] Bilancini S , Lucchi M , Tucci S , Eleuteri P . Functional lymphatic alterations in patients suffering from lipedema. Angiology. 1995;46:333‐339. 7726454 10.1177/000331979504600408

[14] Forner‐Cordero I , Olivan‐Sasot P , Ruiz‐Llorca C , Munoz‐Langa J . Lymphoscintigraphic findings in patients with lipedema. Rev Esp Med Nucl Imagen Mol (Engl Ed). 2018;37:341‐348. 30166264 10.1016/j.remn.2018.06.008

[15] Boursier V , Pecking A , Vignes S . Comparative analysis of lymphoscintigraphy between lipedema and lower limb lymphedema. J Mal Vasc. 2004;29:257‐261. 15738837 10.1016/s0398-0499(04)96770-4

[16] Ma W , Gil HJ , Escobedo N , et al. Platelet factor 4 is a biomarker for lymphatic‐promoted disorders. JCI Insight. 2020;5:e135109. 10.1172/jci.insight.135109 PMC7406300 32525843

[17] Chung NC , Miasojedow B , Startek M , Gambin A . Jaccard/Tanimoto similarity test and estimation methods for biological presence‐absence data. BMC Bioinform. 2019;20:644. 10.1186/s12859-019-3118-5 PMC6929325 31874610

[18] Hissong E , Mowers J , Zhao L , et al. Characterization of the consensus mucosal microbiome of colorectal cancer. NAR Cancer. 2021;3:zcab049. 34988460 10.1093/narcan/zcab049 PMC8693571

[19] Zhao L , Cho WC , Nicolls MR . Colorectal cancer‐associated microbiome patterns and signatures. Front Genet. 2021;12:787176. 35003221 10.3389/fgene.2021.787176 PMC8729777

[20] Veech JA . A probabilistic model for analysing species co‐occurrence. Global Ecol Biogeogr. 2013;22:252‐260.

[21] Faith D , Minchin P , Belbin L . Compositional dissimilarity as a robust measure of ecological distance. Vegetatio. 1987;69:57‐68.

[22] Oksanen J , Blanchet FG, Friendly M, et al. *vegan: Community Ecology Package*. R package version 2.5‐6. 2019.

[23] Mantel N . The detection of disease clustering and a generalized regression approach. Cancer Res. 1967;27:209‐220. 6018555

[24] Paradis E , Schliep K . ape 5.0: an environment for modern phylogenetics and evolutionary analyses in R. Bioinformatics. 2019;35:526‐528. 30016406 10.1093/bioinformatics/bty633

[25] Kassambara A , Mundt F . *factoextra: Extract and Visualize the Results of Multivariate Data Analyses*. R package version 1.0.7. 2020.

[26] Wickham H . ggplot2: Elegant Graphics for Data Analysis. Springer International Publishing; 2016.

[27] Brakenhielm E , Alitalo K . Cardiac lymphatics in health and disease. Nat Rev Cardiol. 2019;16:56‐68. 30333526 10.1038/s41569-018-0087-8

[28] Son A , Jr O'Donnell TF , Izhakoff J , Gaebler JA , Niecko T , Iafrati MA . Lymphedema‐associated comorbidities and treatment gap. J Vasc Surg Venous Lymphat Disord. 2019;7:724‐730. 31248833 10.1016/j.jvsv.2019.02.015

[29] Moni MA , Lio P . How to build personalized multi‐omics comorbidity profiles. Front Cell Dev Biol. 2015;3:28. 26157799 10.3389/fcell.2015.00028 PMC4478898

[30] Goh KI , Cusick ME , Valle D , Childs B , Vidal M , Barabási AL . The human disease network. Proc Natl Acad Sci U S A. 2007;104:8685‐8690. 17502601 10.1073/pnas.0701361104 PMC1885563

[31] de Viron S , Morre SA , Van Oyen H , Brand A , Ouburg S . Genetic similarities between tobacco use disorder and related comorbidities: an exploratory study. BMC Med Genet. 2014;15:85. 25060307 10.1186/1471-2350-15-85 PMC4119471

[32] Islam MM , Valderas JM , Yen L , Dawda P , Jowsey T , McRae IS . Multimorbidity and comorbidity of chronic diseases among the senior Australians: prevalence and patterns. PLoS One. 2014;9:e83783. 24421905 10.1371/journal.pone.0083783 PMC3885451

[33] Chami GF , Kabatereine NB , Tukahebwa EM , Dunne DW . Precision global health and comorbidity: a population‐based study of 16 357 people in rural Uganda. J R Soc Interface. 2018;15. 10.1098/rsif.2018.0248 PMC6228477 30381343

[34] Benn M , Tybjaerg‐Hansen A , Smith GD , Nordestgaard BG . High body mass index and cancer risk‐a Mendelian randomisation study. Eur J Epidemiol. 2016;31:879‐892. 27061578 10.1007/s10654-016-0147-5

[35] Rockson SG . Lymphedema after breast cancer treatment. N Engl J Med. 2019;380:694. 10.1056/NEJMc1817537 30763181

[36] Thomson BR , Heinen S , Jeansson M , et al. A lymphatic defect causes ocular hypertension and glaucoma in mice. J Clin Invest. 2014;124:4320‐4324. 25202984 10.1172/JCI77162 PMC4191022

[37] Nakao S , Hafezi‐Moghadam A , Ishibashi T . Lymphatics and lymphangiogenesis in the eye. J Ophthalmol. 2012;2012:783163. 22523652 10.1155/2012/783163 PMC3317234

[38] Russell PS , Hong J , Windsor JA , Itkin M , Phillips ARJ . Renal lymphatics: anatomy, physiology, and clinical implications. Front Physiol. 2019;10:251. 30923503 10.3389/fphys.2019.00251 PMC6426795

[39] Mortimer PS , Rockson S . New developments in clinical aspects of lymphatic disease. J Clin Invest. 2014;124:915‐921. 24590276 10.1172/JCI71608 PMC3938261

[40] Helyer LK , Varnic M , Le LW , Leong W , McCready D . Obesity is a risk factor for developing postoperative lymphedema in breast cancer patients. Breast J. 2010;16:48‐54. 19889169 10.1111/j.1524-4741.2009.00855.x

[41] Mehrara BJ , Greene AK . Lymphedema and obesity: is there a link? Plast Reconstr Surg. 2014;134:154e‐160e. 10.1097/PRS.0000000000000268 PMC4393748 25028830

[42] Greene AK , Grant FD , Slavin SA , Maclellan RA . Obesity‐induced lymphedema: clinical and lymphoscintigraphic features. Plast Reconstr Surg. 2015;135:1715‐1719. 25724063 10.1097/PRS.0000000000001271

[43] Randolph GJ , Ivanov S , Zinselmeyer BH , Scallan JP . The lymphatic system: integral roles in immunity. Annu Rev Immunol. 2017;35:31‐52. 10.1146/annurev-immunol-041015-055354 PMC5551392 27860528

[44] Pasupneti S , Tian W , Tu AB , et al. Endothelial HIF‐2alpha as a key endogenous mediator preventing emphysema. Am J Respir Crit Care Med. 2020;202(7):983‐995. 32515984 10.1164/rccm.202001-0078OC PMC7528783

[45] Botros L , Vonk Noordegraaf A , Aman J . Vanishing vessels aboding pulmonary disease: a role for VEGFR2. Eur Respir J. 2020;55:2000326. 10.1183/13993003.00326-2020 32245777

[46] Morgan AD , Sharma C , Rothnie KJ , Potts J , Smeeth L , Quint JK . Chronic obstructive pulmonary disease and the risk of stroke. Ann Am Thorac Soc. 2017;14:754‐765. 28459623 10.1513/AnnalsATS.201611-932SR PMC5427743

[47] Rasmussen JC , Aldrich MB , Tan IC , et al. Lymphatic transport in patients with chronic venous insufficiency and venous leg ulcers following sequential pneumatic compression. J Vasc Surg Venous Lymphat Disord. 2016;4:9‐17. 26946890 10.1016/j.jvsv.2015.06.001 PMC4782606

[48] Sweeney MD , Zlokovic BV . A lymphatic waste‐disposal system implicated in Alzheimer's disease. Nature. 2018;560:172‐174. 30076374 10.1038/d41586-018-05763-0 PMC6201839

[49] Moriguchi P , Sannomiya P , Lara PF , Oliveira‐Filho RM , Greco KV , Sudo‐Hayashi LS . Lymphatic system changes in diabetes mellitus: role of insulin and hyperglycemia. Diabetes Metab Res Rev. 2005;21:150‐157. 15386809 10.1002/dmrr.500

