# Supplementary figures and images for: Exploring disease interrelationships in patients with lymphatic disorders: A single center retrospective experience
Source: Clin Transl Med. 2022 Apr 22;12(4):e760. doi: 10.1002/ctm2.760 (PMC9028099; doi:10.1002/ctm2.760)

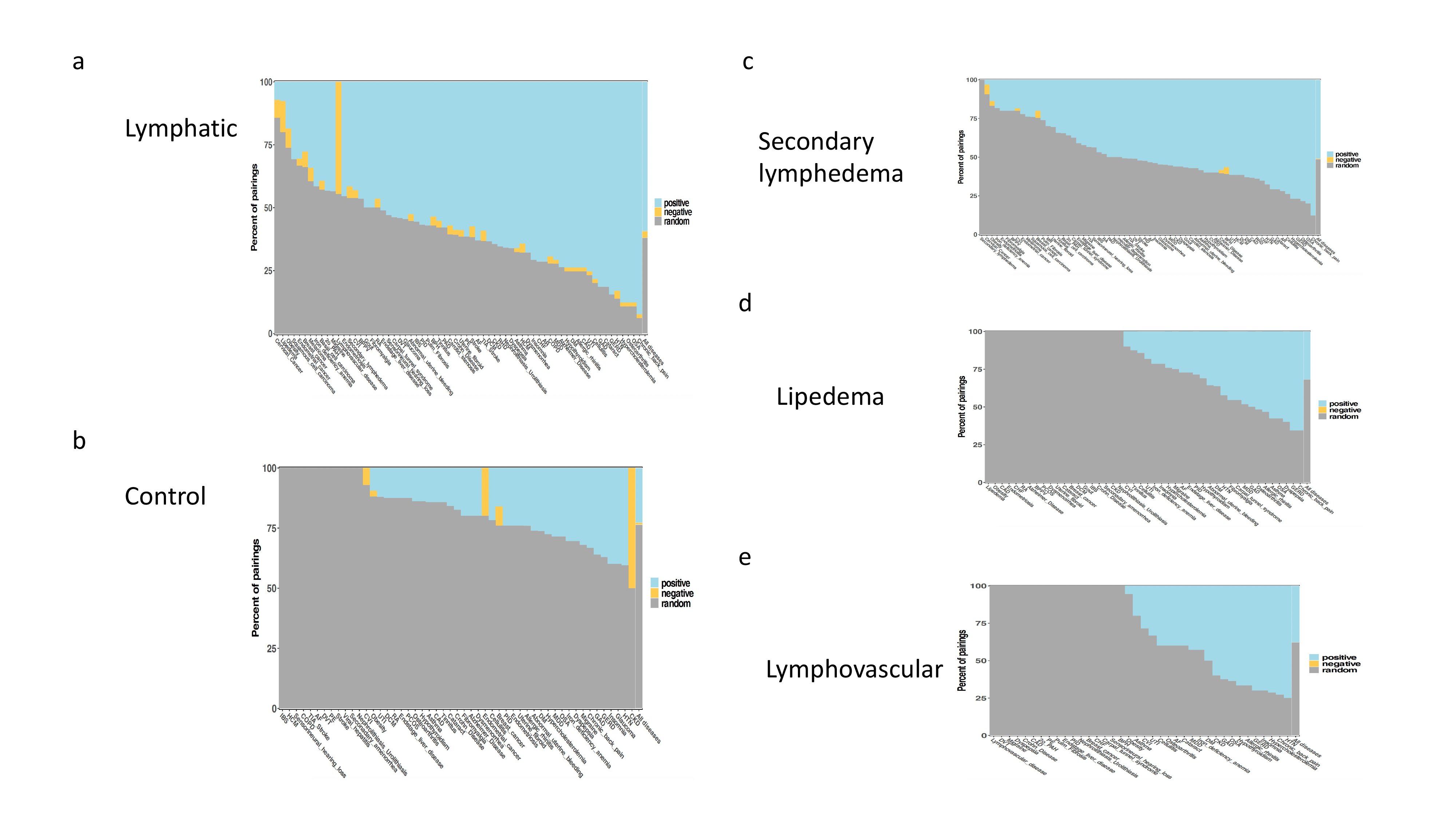

Supplement: Supplementary file 1 — Supplementary Figure 1. Disease interrelationships analysed by pairwise co‐occurrences. [file CTM2-12-e760-s008.tif]

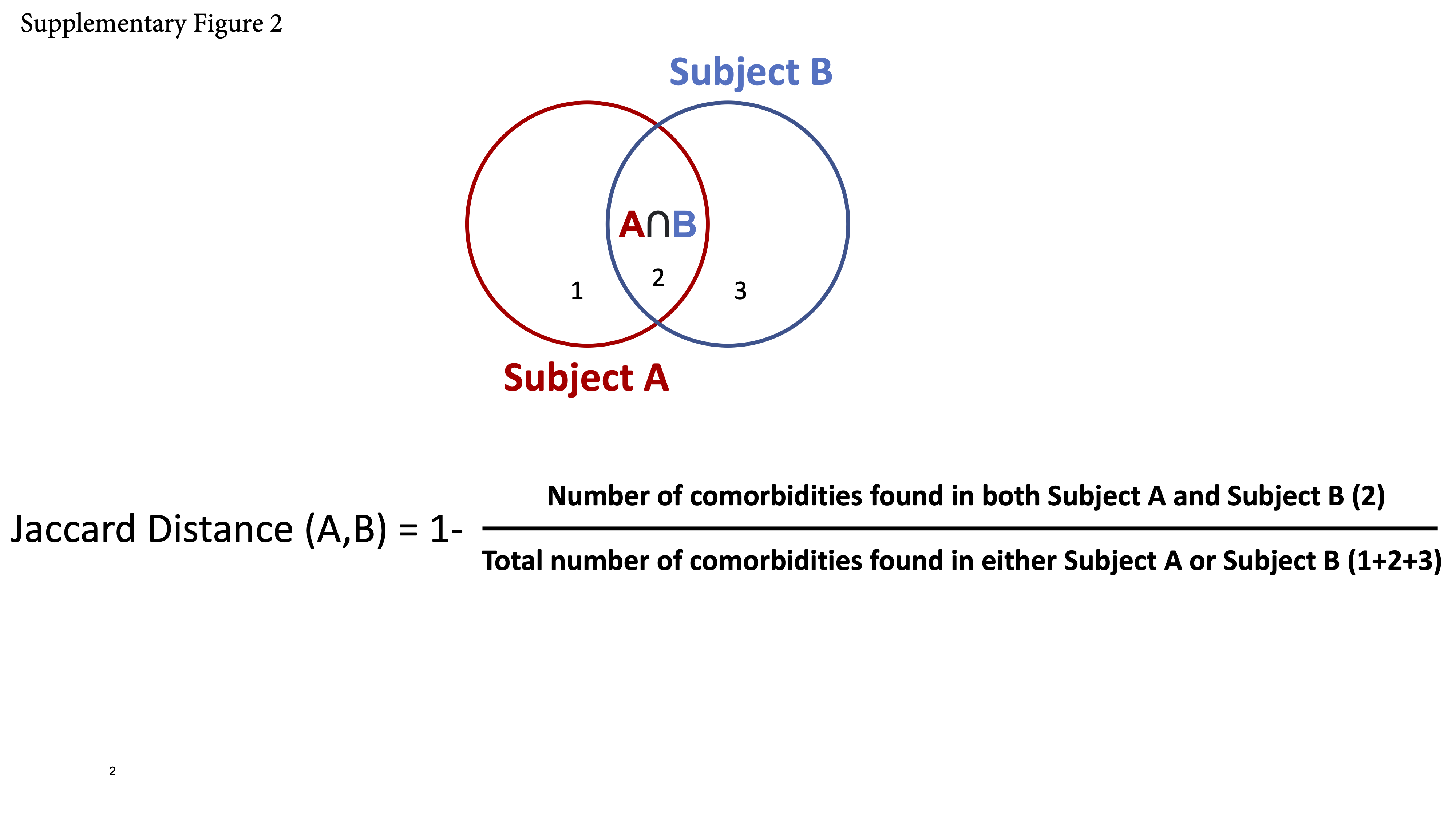

Supplement: Supplementary file 2 — Supplementary Figure 2. Schematic representation of the Jaccard distance calculation as a Venn diagram. The distance between subjects A and B is depicted as one minus the total number of diseases found in both subjects (segment 2 of the Venn Diagram) divided by the total number of diseases found in the two subjects (segments 1 + 2 + 3 in the Venn Diagram). [file CTM2-12-e760-s006.tiff]

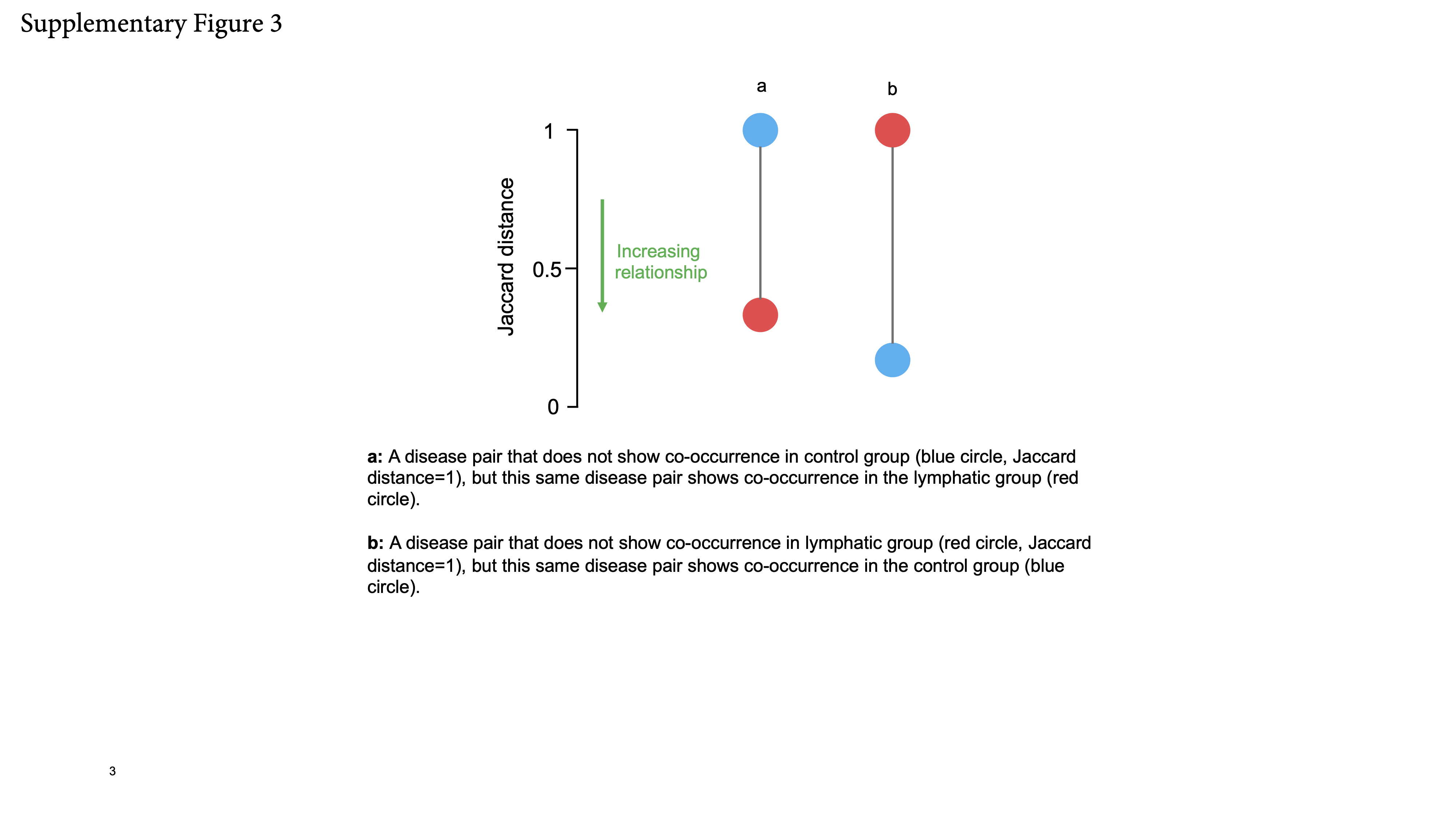

Supplement: Supplementary file 3 — Supplementary Figure 3. Schematic representation of Jaccard distance analysis. As seen in Figure 2 in the text, Jaccard distances of control group are depicted in blue and Jaccard distances in lymphatic groups are shown in red. A value of 1 (blue) indicates that there is no co‐occurrence of the depicted disease pair in the non‐L cohort. The length of the line that connects the blue circle to the red (value < 1) is representative of the likelihood of co‐occurrence of the disease pair within the L cohort; an increasing length of the line indicates an increased likelihood of co‐occurrence. A similar display can be created for the disease pairs in which the Jaccard distance = 1 in the lymphatic cohorts (red), but co‐occur in the control cohorts (blue circle, value < 1), thereby permitting analysis of those disease pairs that have a decreased likelihood of co‐occurrence in the setting of a lymphatic diagnosis. [file CTM2-12-e760-s009.tiff]
