# Supplementary material for: Exploring disease interrelationships in patients with lymphatic disorders: A single center retrospective experience
Source: Clin Transl Med. 2022 Apr 22;12(4):e760. doi: 10.1002/ctm2.760 (PMC9028099; doi:10.1002/ctm2.760)
Supplement: Supplementary file 4 — Figures [file CTM2-12-e760-s004.pdf]

Supplementary Figure 1

a

Control

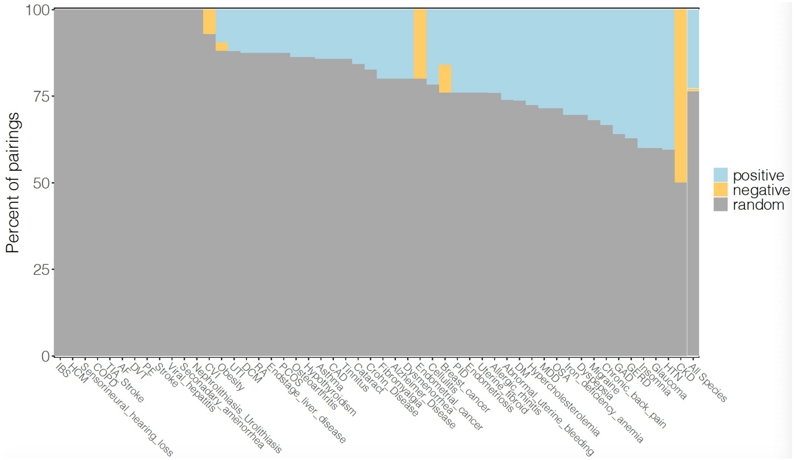

b

Lymphatic

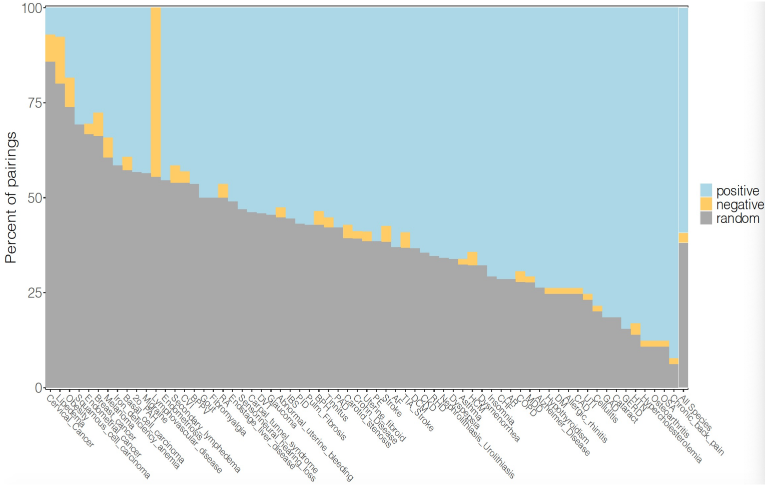

c

Secondary lymphedema

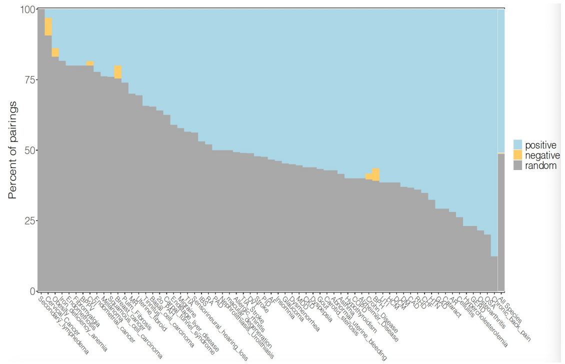

d

Lipedema

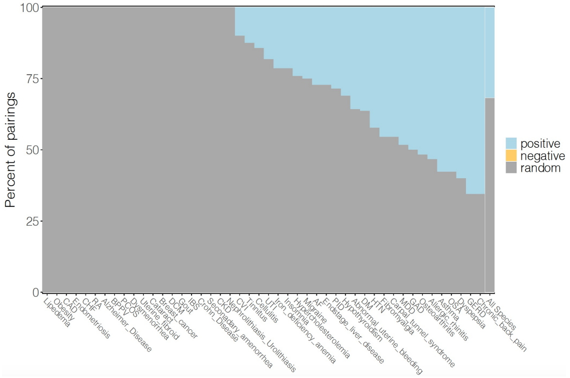

e

Lymphovascular

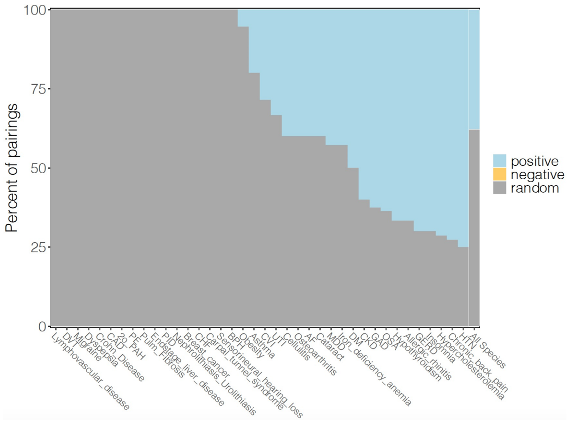

Supplementary Figure 2

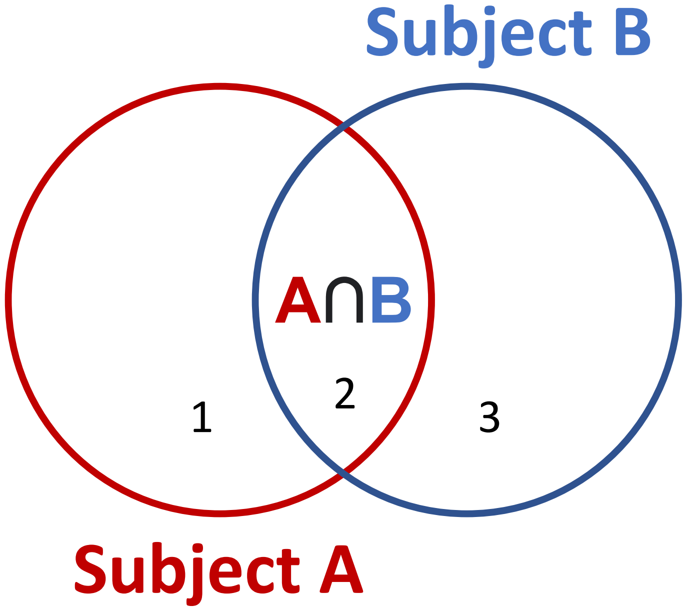

$$\text{Jaccard Distance (A,B)} = 1 - \frac{\textbf{Number of comorbidities found in both Subject A and Subject B (2)}}{\textbf{Total number of comorbidities found in either Subject A or Subject B (1+2+3)}}$$

Supplementary Figure 3

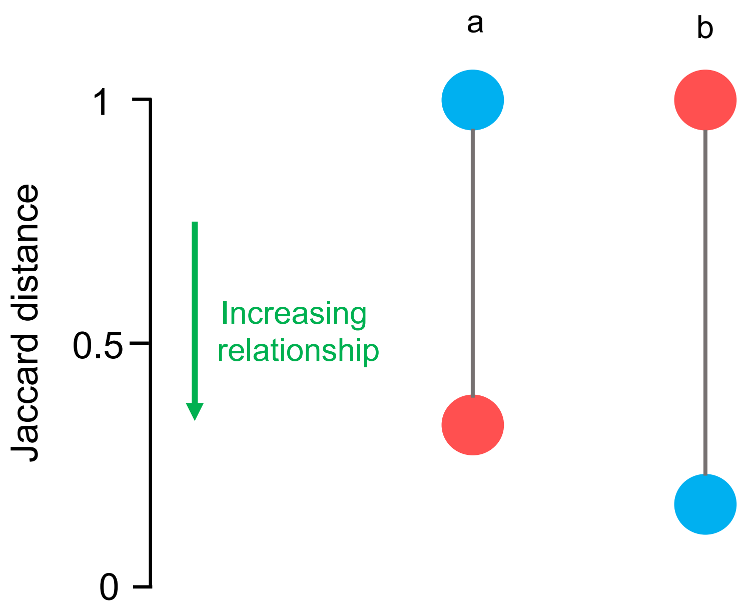

a: A disease pair that does not show co-occurrence in control group (blue circle, Jaccard distance=1), but this same disease pair shows co-occurrence in the lymphatic group (red circle).

b: A disease pair that does not show co-occurrence in lymphatic group (red circle, Jaccard distance=1), but this same disease pair shows co-occurrence in the control group (blue circle).
